# Microbial glycolipoprotein-capped silver nanoparticles as emerging antibacterial agents against cholera

**DOI:** 10.1186/s12934-016-0422-x

**Published:** 2016-02-01

**Authors:** Geeta Gahlawat, Sristy Shikha, Baldev Singh Chaddha, Saumya Ray Chaudhuri, Shanmugam Mayilraj, Anirban Roy Choudhury

**Affiliations:** CSIR-Institute of Microbial Technology, Sector 39A, Chandigarh, 160036 India

**Keywords:** *Ochrobactrum rhizosphaerae*, Glycolipoprotein, Silver nanoparticles, Antibacterial agent, XTT assay, *Vibrio cholerae*

## Abstract

**Background:**

With the increased number of cholera outbreaks and emergence of multidrug resistance in *Vibrio cholerae* strains it has become necessary for the scientific community to devise and develop novel therapeutic approaches against cholera. Recent studies have indicated plausibility of therapeutic application of metal nano-materials. Among these, silver nanoparticles (AgNPs) have emerged as a potential antimicrobial agent to combat infectious diseases. At present nanoparticles are mostly produced using physical or chemical techniques which are toxic and hazardous. Thus exploitation of microbial systems could be a green eco-friendly approach for the synthesis of nanoparticles having similar or even better antimicrobial activity and biocompatibility. Hence, it would be worth to explore the possibility of utilization of microbial silver nanoparticles and their conjugates as potential novel therapeutic agent against infectious diseases like cholera.

**Results:**

The present study attempted utilization of *Ochrobactrum rhizosphaerae* for the production of AgNPs and focused on investigating their role as antimicrobial agents against cholera. Later the exopolymer, purified from the culture supernatant, was used for the synthesis of spherical shaped AgNPs of around 10 nm size. Further the exopolymer was characterized as glycolipoprotein (GLP). Antibacterial activity of the novel GLP–AgNPs conjugate was evaluated by minimum inhibitory concentration, XTT reduction assay, scanning electron microscopy (SEM) and growth curve analysis. SEM studies revealed that AgNPs treatment resulted in intracellular contents leakage and cell lysis.

**Conclusion:**

The potential of microbially synthesized nanoparticles, as novel therapeutic agents, is still relatively less explored. In fact, the present study first time demonstrated that a glycolipoprotein secreted by the *O. rhizosphaerae* strain can be exploited for production of AgNPs which can further be employed to treat infectious diseases. Although this type of polymer has been obtained earlier from marine fungi and bacteria, none of these reports have studied the role of this polymer in AgNPs synthesis and its application in cholera therapy. Interestingly, the microbial GLP-capped AgNPs exhibited antibacterial activity against *V. cholerae* comparable to ciprofloxacin. Thus the present study may open up new avenues for development of novel therapeutic agents for treatment of infectious diseases.

**Graphical abstract:**
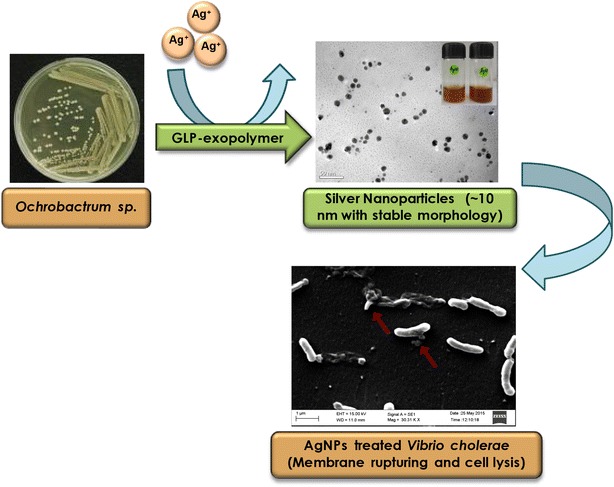
Development of novel therapeutic agents for treatment of cholera

## Background

Nanoscale materials have generated a lot of interest among researchers due to various anticipated applications in different areas such as biomedical, pharmaceutical, electronics, catalysis and biosensors [1]. Recently, metallic nanoparticles have received significant attention in biomedical field for the treatment of various acute infectious diseases such as cholera. Among the noble metallic nanoparticles, Silver nanoparticles (AgNPs) are the most important candidates of choice for solving various medical problems due to their chemical inertness, biocompatibility, oxidation resistance and wide spectrum of antimicrobial activity against diverse range of bacteria and fungi [2]. In addition, silver nanoparticles have very little chance of generating drug resistant microbial strains. Therefore, AgNPs have emerged as effective antimicrobial agents to treat infectious diseases caused by human pathogens and eliminate multidrug resistance problem [3]. However, all medical applications require biocompatible structures, which inspired scientists to adopt green synthetic routes to fabricate nanostructures [4, 5].

In recent times, facile and green synthesis of different metal nanostructures has drawn remarkable attention from researchers. Green synthesis refers to the recruitment of biogenic matter including plant extracts, biopolymers and microbial sources like bacteria, fungi, algae, yeast for nanomaterials fabrication [2]. Biologically synthesized nanoparticles are free of toxic chemicals and hazardous by-products in contrary to their physical and chemically synthesized counterparts. Amongst different green routes, microbial systems have been employed extensively for facile synthesis of AgNPs owing to its ease of manipulations [4]. Microbes have an innate tendency to reduce toxic metal ions into clean inert metal particles as a part of their defense system against metal toxicity in their vicinity [6]. Microbes comprise of a versatile microenvironment which may provide reducing and stabilizing agents for metal nanoparticles thus helping these ubiquitous organisms to transform into nanofactories. Extracellular synthesis of AgNPs using cell-free culture broth of microorganisms could be a simple, advantageous and cost effective approach as it minimizes the downstream processing steps [7, 8]. Although some literature reports have demonstrated synthesis of AgNPs using microbial culture broth [9, 10], the exact bioactive moiety and mechanism responsible for AgNP synthesis is not yet clear.

The present study aimed to develop a novel bioconjugate for cholera treatment via a green route. In the present study, a novel bacterial strain ARC-61 was characterized as *Ochrobactrum rhizosphaerae* which was further used for the synthesis of smaller sized spherical silver nanoparticles. An exopolymer (EPL) secreted from this bacterium later characterized as glycolipoprotein, was found to be responsible for biomineralization of silver ions. This is the first report on the preparation of AgNPs by reduction and stabilization of the corresponding metal salts using microbial glycolipoprotein polymer. The antibacterial property of the synthesized AgNPs was tested against *Vibrio cholerae*, a causative agent of life-threatening secretory diarrheal disease. Cholera is an acute infectious disease that can kill within hours if left untreated. It remains the second most common cause of death particularly in developing countries. There are around 3–5 million cases that occur globally every year and about 10,000–120,000 people die [11]. At present oral rehydration therapy is the mainstay for cholera, it is therefore essential to design better therapeutic approaches against cholera, including new antibiotics to treat this deadly disease. The prospect for developing new generation antibiotics and unique mode of action against microbes makes AgNPs an attractive alternative to antibiotics to overcome the multi-drug resistance problem. Thus the antibacterial property of synthesized AgNPs was evaluated against *V. cholerae* using metabolic activity assay and growth curve analysis, and compared with the ciprofloxacin antibiotic. To assess the antibacterial activity, the minimum inhibitory concentration (MIC) of AgNPs and bacterial cell viability were determined.

## Results and discussions

### Strain characterization

**Fig. 1:**
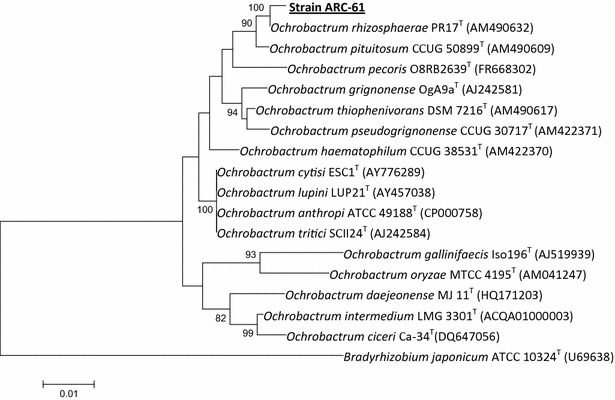
Neighbour-joining tree based on 16S rRNA gene sequences showing relationships of isolate ARC-61 with closely related species of the genus *Ochrobactrum*. *Bar* 0.01 indicates substitutions per nucleotide position. *Bradyrhizobium japonicum* (ATCC 10324^T^) was used as an out-group. *Bootstrap* values (>70 %) based on 100 re-sampled datasets are shown at branch nodes

The 16S rRNA gene sequence of strain ARC-61 was aligned with sequences of other species of genus *Ochrobactrum* retrieved from GenBank data base. The strain showed highest degree of similarity with *Ochrobactrum rhizosphaerae* (99.71 %) followed by *Ochrobactrum thiophenivorans* (98.74 %), *Ochrobactrum pituitosum* (98.74 %), *Ochrobactrum grignonense* (98.62 %) and *Ochrobactrum anthropi* (98.28 %). Based on the phylogenetic analysis and the comparison of biochemical test results with type strain of *Ochrobactrum rhizosphaerae* (Cells of strain ARC-61 are Gram-negative short rods; positive for utilization of d-fructose, l-rhamnose, sodium citrate, maltose, d-sorbitol and negative for adonitol, cellobiose, gentibiose and raffinose; positive for oxidase production and tween 80 hydrolysis), the strain ARC-61 has been identified as *O. rhizosphaerae* (Fig. 1).

### Biosynthesis and characterization of AgNPs from culture broth

**Fig. 2:**
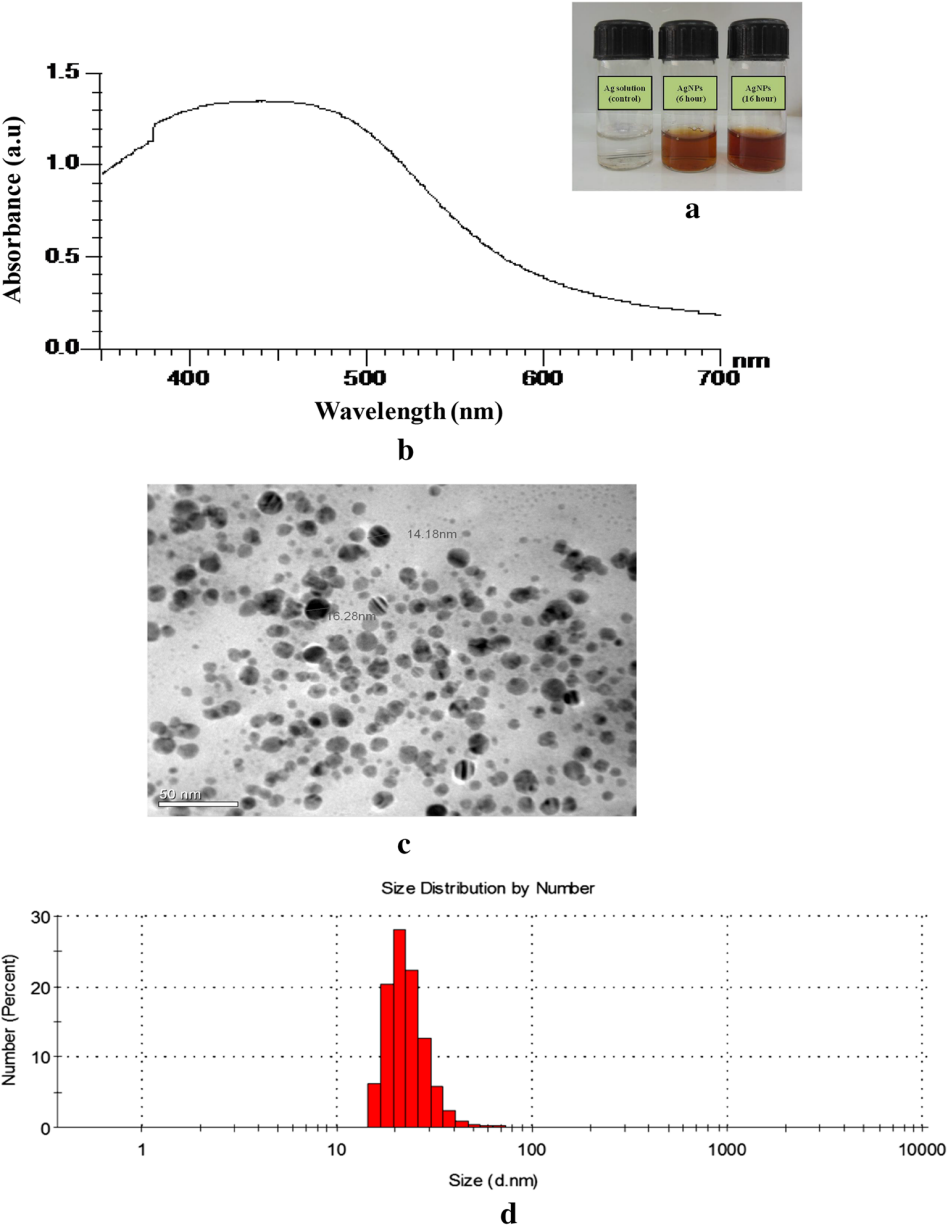
**a** Synthesis of AgNPs by culture broth at 37 °C. Change in color of AgNO_3_ solution from colorless to dark brown after 24 h. **b** UV–visible spectra of AgNPs synthesized via supernatant (SN). **c** TEM micrograph of SN–AgNPs. *Scale bars* correspond to 50 nm. **d** DLS *histogram* of SN–AgNPs indicates size distribution by number

The formation of AgNPs using supernatant (SN) was checked by visual examination of the reaction mixture by a color change from colorless to yellowish brown (Fig. 2a). In contrast, the control silver nitrate solution without supernatant showed no color change. Further to support visual examination, morphological and quantitative analysis was carried out by UV–visible spectrophotometer (Fig. 2b), transmission electron microscopy (TEM) and dynamic light scattering (DLS). TEM graphs demonstrated the formation of nanoparticles within a size range of 5–25 nm (Fig. 2c). The DLS histogram indicated that the average particle size of SN–AgNPs was around 21 nm (Fig. 2d), which was in accordance with the data obtained by TEM imaging.

### Isolation and characterization of exopolymer (EPL)

Extracts obtained from biological organisms such as microbe and plant sources may act as both reducing and capping agents and have been exploited extensively. However, majority of the literature reports were unable to find out the active biomolecule involved in AgNP synthesis. The purification of active biocomponent from biological complex mixtures and a detailed knowledge about this component in biosynthesis of AgNPs will help in producing homogeneous tailor made solution of AgNPs. Moreover, this will surely enhance the possibility of commercialization of AgNPs production. Most of the earlier studies hypothesized that AgNP production through biological agents require involvement of nitrate reductase enzyme [12–14]. In contrary to the earlier reports, in the present study the active biomolecule responsible for synthesis and capping of silver nanoparticles was clearly identified. The bioactive molecule was extracted and purified from culture supernatant for further mechanistic evaluation of the silver nanoparticle synthesis. Further characterization indicated it as a glycolipoprotein exopolymer (EPL). Purified exopolymer (EPL) mediated AgNPs synthesis resulted in very small size nanoparticles with narrow size distribution. Preliminary characterization of the exopolymer using various biophysical and chemical techniques indicated that it is a mixture of three major components namely carbohydrates (34.8 %), lipids (33 %) and protein (29 %). Thus the observation suggested that the EPL responsible for the reduction and stabilization of silver nanoparticle is actually a glycolipoprotein (GLP). Earlier, this type of polymer has been obtained from marine endosymbiotic fungi, *Aspergillus ustus* and marine bacterial culture, *Oceanobacillus* [15, 16]. However none of the earlier report has studied the role of microbial GLP polymer in AgNPs synthesis and its application in cholera therapy.

### Antioxidant activity

**Fig. 3:**
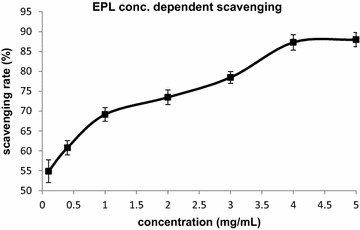
DPPH radical scavenging activity of GLP polymer. Each value represents the average of three experiments. *Error bars* represent standard deviation

To further substantiate the results of GLP acting as a functional moiety in capping and stabilization of AgNP, the reducing ability (or antioxidant property) of GLP was investigated by 2,2-diphenyl-1-picrylhydrazyl (DPPH) assay. The DPPH is a stable nitrogen-centered free radical and can be reduced by accepting an electron or hydrogen in the presence of an antioxidant. It has been widely employed to investigate the free radical scavenging and hydrogen donation properties of antioxidants compounds [17]. All the samples demonstrated scavenging activity on DPPH radical in a concentration-dependent manner (Fig. 3). However the scavenging activity did not increase after the 4 mgmL^−1^concentration of the GLP. The 4 mgmL^−1^GLP solution showed stronger DPPH scavenging activity than other samples. The effect of antioxidants on DPPH scavenging was influenced by many factors and some literature reports have suggested that hydroxyl (–OH) group of monosaccharide units can donate proton to combine with unpaired electron of DPPH to form non-radical DPPH–H [18]. Hence, it may be commented, that GLP was the main component of the culture broth responsible for silver biomineralization.

### Quantification and characterization of AgNPs synthesized via GLP polymer

**Fig. 4:**
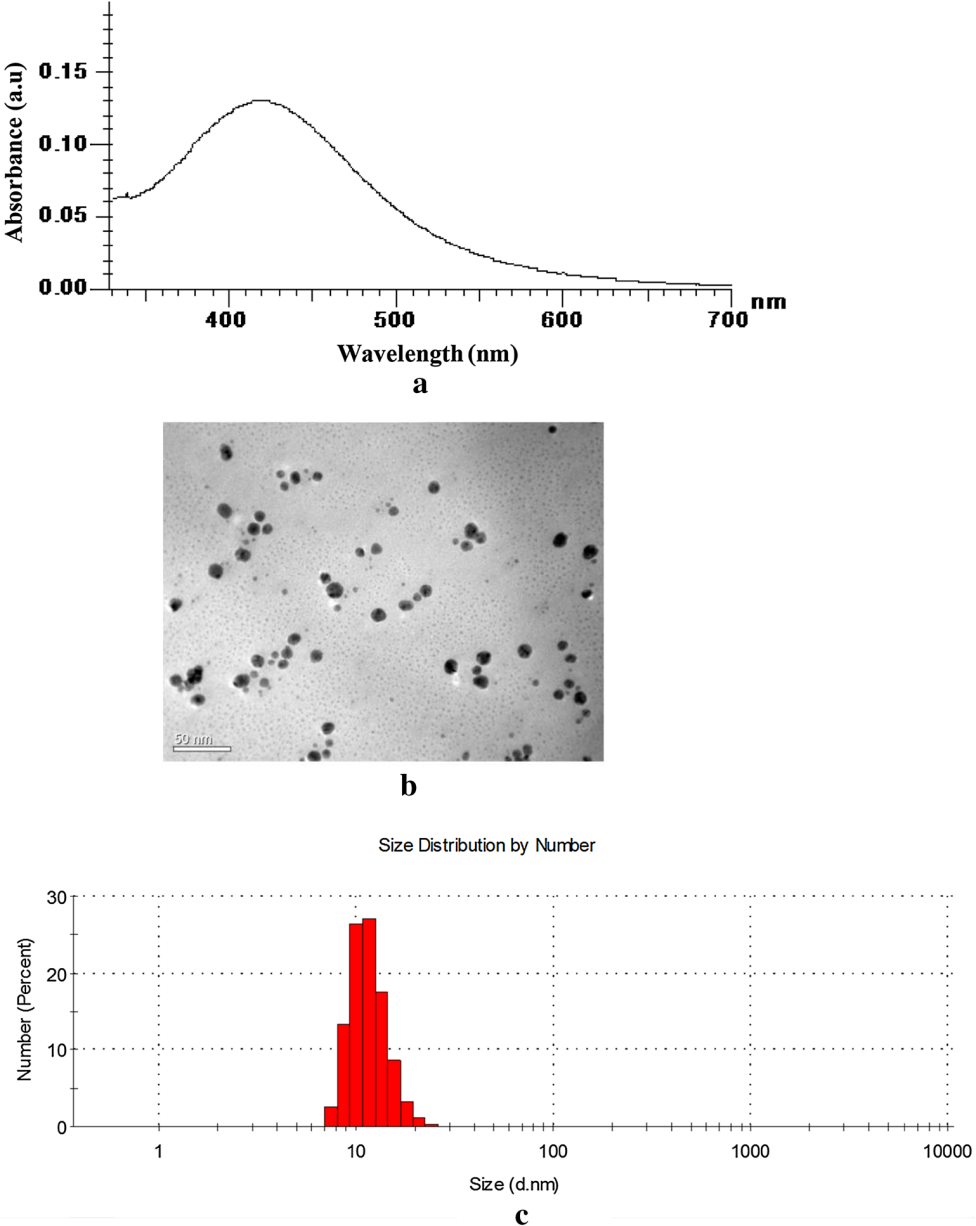
**a** Characteristic surface plasmon resonance (SPR) of AgNPs synthesized via GLP. **b** TEM micrograph of GLP-AgNPs. *Scale bars* correspond to 50 nm. **c** DLS *histogram* of GLP–AgNPs indicates size distribution by number

The reduction of Ag ions by GLP and biosynthesis of AgNPs was confirmed by UV–vis absorption spectroscopy. A wavelength scan in the UV–vis spectra demonstrated characteristic absorption maxima at 420 nm due to surface plasmon resonance (SPR) transfer of AgNPs (Fig. 4a). Earlier reports [8, 19] have obtained similar results and shown that a characteristic absorption maximum for AgNPs occurs at 420 nm. The stability of same AgNP solution was determined by measuring its UV–vis absorption spectra after 30 days and no significant change in the absorption spectra was observed. The absorption maximum was obtained at 425 nm which was almost similar to the initial peak position indicating no agglomeration of AgNPs. This phenomenon revealed that the AgNPs were well capped and stabilized by GLP macromolecules. The molar concentration and numbers of spherical AgNPs were calculated by UV–visible spectra data as follows [20]:

1 $$A = c. \varepsilon .do$$

2 $$c =N/ {N_{A}}$$

where, *A* is the absorption maxima at 420 nm, *ε* is molar decadic extinction coefficient of AgNPs with diameter *d*, *c* is molar concentration of NPs, *do* is the path length of light, *N* and *N*_*A*_ is the number of NPs and Avogadro constant, respectively.

### Morphological study using TEM and DLS

TEM analysis of GLP–AgNPs elucidated formation of spherical-shaped particles well segregated from each other. TEM analysis at different magnifications displayed particles within a size range of 2–20 nm (Fig. 4b). In literature, similar observations have been reported by other investigators using starch and dextran [21, 22]. DLS histogram indicated most of the particles were in the size range of 5–20 nm which was in well agreement with the data obtained by TEM imaging. However, the average particle size of AgNPs was found to be 10 nm (Fig. 4c). Earlier reports on AgNPs synthesis using EPL indicated formation of larger size nanoparticles, [5, 23] however, in the present report it was possible to obtain monodisperse suspension of AgNPs having smaller size nanoparticle with narrow particle size distribution suggesting that GLP polymer has a better reducing and stabilizing properties and may be successfully used for biomimetic synthesis of AgNPs.

### Energy dispersive X-ray analysis (EDX) and FT-IR spectra analysis

**Fig. 5:**
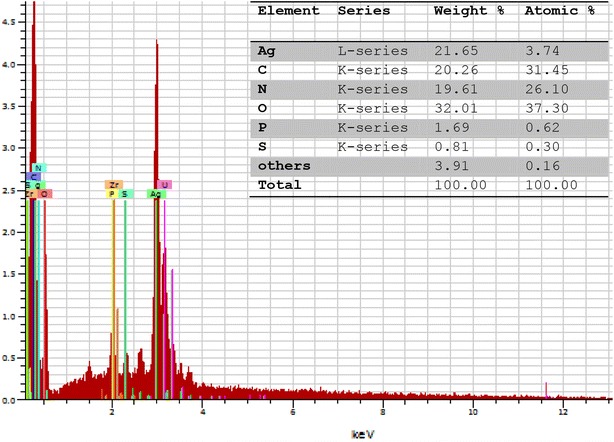
EDX spectrum of AgNPs stabilized by GLP molecule

The energy dispersive X-ray analysis (EDX) of GLP–AgNPs has revealed strong signal in the silver region, and thus confirmed the formation of silver nanoparticles. The EDX spectrum showed the strongest absorption peak at ~3 keV due to surface plasmon resonance by silver (Ag) atoms (Fig. 5). Moreover, C, O and N atoms showed relatively similar intensity to that of elemental Ag. The EDX pattern of GLP stabilized AgNPs consisted of 21.65 wt % Ag, 20.26 wt % C, 19.61 wt % N and 32.01 wt % O (Fig. 5 inset). Similar results were observed by Kanmani and Lim [1] who reported that the EDX spectrum of exopolysaccharide-stabilized silver nanoparticles had the strongest peaks at ~3 keV, confirming the presence of elemental Ag in the nanoparticles.

**Fig. 6:**
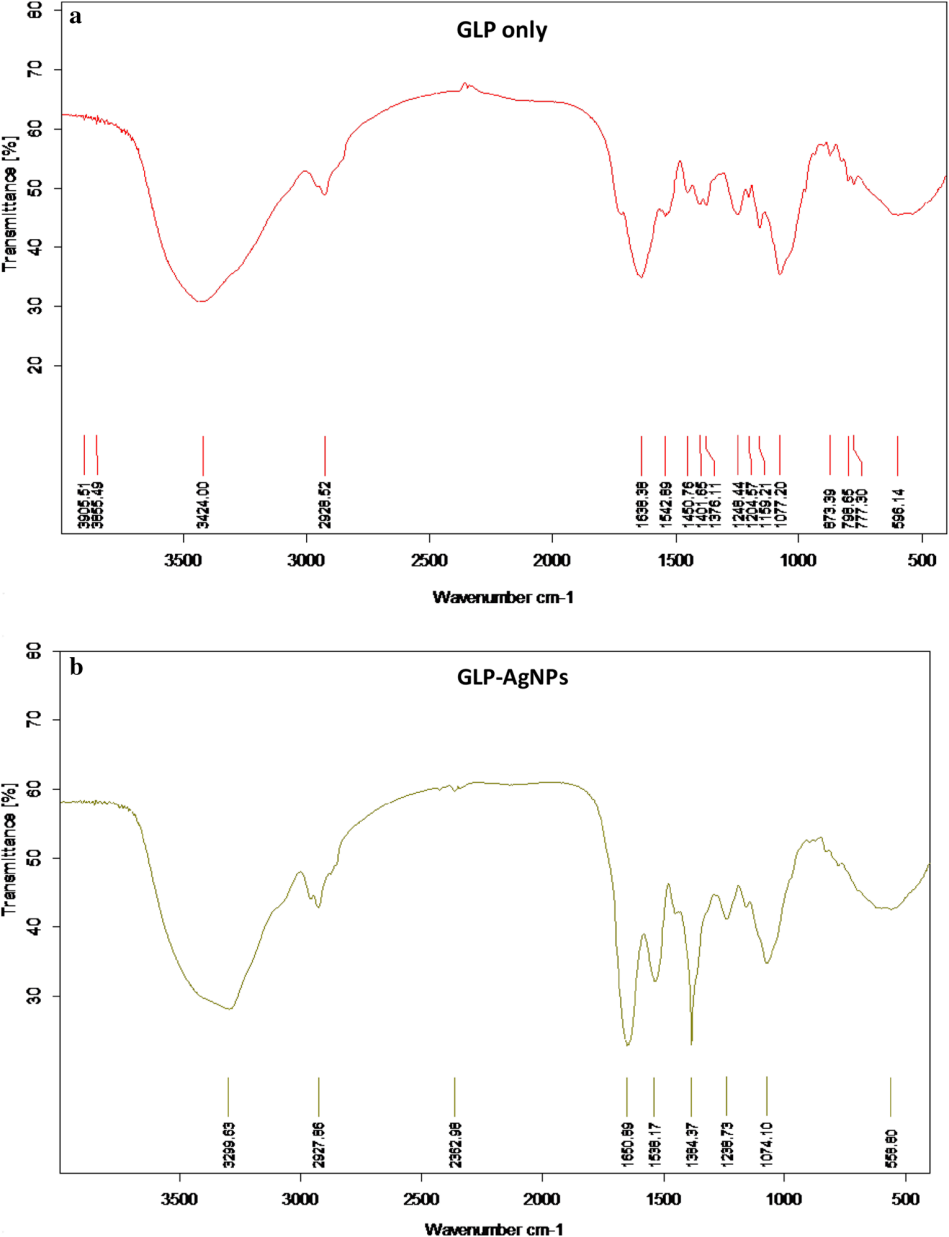
FT-IR spectra of **a** GLP only and **b** GL–AgNPs

FT-IR spectra of pure GLP and GLP based silver nanoparticles were studied to further gain an insight towards the biomineralization process and to investigate the role of various functional groups of GLP involved in reduction and stabilization of AgNPs (Fig. 6). The spectrum provided an impression towards the involvement of various functional groups responsible for the bio-reduction of metal ions to nanocolloidal solution. FT-IR spectra of both samples clearly represent various characteristic peaks between 3650 and 750 cm^−1^ (Fig. 6a, b). FTIR spectra of both sample displayed strong absorption at 3424 cm^−1^ which may be attributed to stretching vibration of O–H group. In both spectra, an adsorption peak was observed at 2928 cm^−1^ which correspond to asymmetrical C–H stretching vibration [1]. Additionally, a sharp absorption peak at 1638 cm^−1^ demonstrated stretching vibration due to carbonyl group (C = O). The characteristic absorption peaks of GLP were observed between 1070 and 1450 cm^−1^ in FTIR spectrum of both pure GLP and GLP mediated AgNPs. This observation clearly elucidated that the basic structure of GLP remained unchanged during AgNPs synthesis. However, minor shifting of the absorption peaks were observed in case of GLP based silver nanoparticles spectra as compared to the pure GLP spectra. This shifting of the peaks may be due to the strong interaction of Ag^+^ with GLP functional groups [1]. Thus, it may be hypothesized that the free CH_2_OH groups of pure GLP molecules were oxidized to carboxyl groups (COO^−^), while subsequent reduction of Ag^+^ to Ag^0^ leading to the formation of silver nanoparticles [5]. Interestingly, an increase in the intensity of absorption peak was observed at 1538 cm^−1^ when GLP–AgNPs spectrum was compared with pure GLP spectrum. This absorption may be attributed to symmetric stretching vibrations of free carboxylate groups [24] of amino acid residues in the protein. An additional absorption peaks at 1384 cm^−1^ have originated due to C–N stretching vibrations of amino group which suggested the role of protein moieties present in the GLP towards the fabrication as well as the stabilization of metal nanoparticles [10]. Similar observation has also been made by Kaler et al. (2012) and indicated the role of protein in biomineralization of silver ions [10].

### Antibacterial activity of AgNPs against *V. cholerae*

**Fig. 7:**
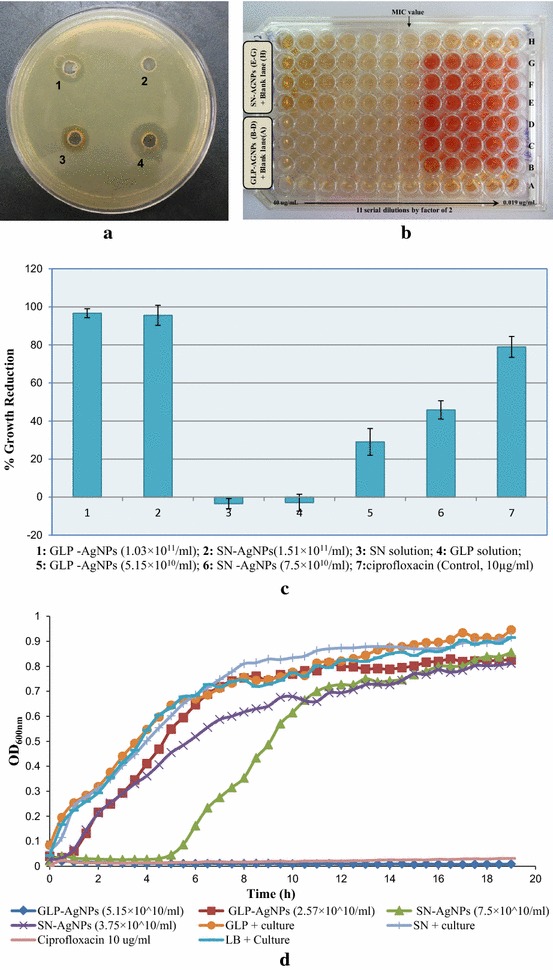
**a** Zone of inhibition of AgNPs against *Vibrio cholerae* 1. (*SN–AgNPs*, 2. *Supernatant*, *Silver nitrate* (AgNO_3_), *GLP–AgNPs*). **b** Microtitre plate demonstrates MIC value (no color change in first 7 wells) using XTT assay. *Lane* B–D—GLPAgNPS; *Lane* E–G—SNAgNPs; *Lane* A and H—LB broth (Blank) **c** Antibacterial activity of GLP–AgNPs and SN–AgNPs against *V. cholerae* and MIC values for growth as determined by XTT-reduction assay. **d** Growth kinetics of *V. cholerae* in LB (negative control), with GLP and SN alone, with ciprofloxacin (positive control), with GLP–AgNPs and SN–AgNPs at two-fold and four-fold lower concentration than MICs

The preliminary agar diffusion assay elucidated that the as-synthesized AgNPs had antibacterial activity against the *V. cholerae* N16961. After 16 h of incubation, a clear zone of bacterial inhibition was observed in wells loaded with AgNPs, whereas control well loaded with phosphate buffer saline (PBS) alone did not exhibit any inhibition zone. Inhibition zones of around 15 mm and 11 mm were obtained by GLP–AgNPs and SN–AgNPs, respectively. Although AgNO_3_ solution also inhibited the growth, the zone of inhibition (~7 mm) was much smaller as compared to the AgNPs (Fig. 7a). In general, GLP–AgNPs exhibited a slightly higher antimicrobial activity compared to SN–AgNPs as demonstrated by greater zone of inhibition by GLP–AgNPs.

Antibacterial activity of the AgNPs synthesized by GLP and SN was further assayed against the human pathogen, *V. cholerae* N16961 by minimal inhibitory concentration (MIC) and XTT [2,3-bis(2-methoxy-4-nitro-5-sulfophenyl)-2H-tetrazolium-5-carboxanilide sodium salt] assay. MIC is defined as the lowest concentration at which no increase in the absorbance of the bacteria was observed. However, as the measurement of turbidity cannot distinguish between live and dead bacteria, hence bacterial viability was tested by a colorimetric XTT-reduction assay, which allows detection of viable bacteria by their metabolic activity [25].The MIC in the XTT assay was defined as the lowest concentration of AgNPs that prevented color change as described by McCluskey et al. (2005) [26]. Both AgNPs demonstrated reproducible antibacterial activity against *V. cholerae* N16961 using XTT assays (Fig. 7b).

In general, GLP–AgNPs concentration of 1.03 × 10^11^ NP per ml was sufficient to completely inhibit and kill the *V. cholerae*, indicating that the MIC value of GLP–AgNPs against *V. cholerae* was 1.03 × 10^11^ NPml^−1^ (Fig. 7c). Similarly in case of SN–AgNPs, the MIC value was found to be 1.51 × 10^11^ NPml^−1^. One way analysis of variance (ANOVA) was applied on the determined MIC values of AgNPs, controls (SN and GLP) and ciprofloxacin. ANOVA of MIC assay for *V.cholerae* demonstrated that the ‘*F*’ and ‘*P*’ value were 243.15 and <0.001 respectively, which indicated that the differences among the results were statistically significant. This analysis showed significant change of inhibitory concentration range among AgNPs, controls and antibiotic. Furthermore, the as synthesized AgNPs demonstrated bactericidal effects by not only inhibiting the bacterial growth but also reducing the metabolic activity and cell viability of bacteria. This irreversible inhibition of bacterial growth has been reported to be desirable to prevent bacterial colonization of silver-containing medical devices, such as catheters where bacteria-killing activity is required [23].

### Anti-cholera activity of the GLP-capped AgNPs

The ability of the GLP-capped AgNPs to inhibit the growth and its effect on cell morphology was studied. Complete growth inhibition was observed in the presence of GLP–AgNPs and SN–AgNPs at MICs. Growth kinetic pattern in case of AgNPs at MIC was exactly similar to ciprofloxacin (10 μgmL^−1^), the standard antibiotic. Moreover, the growth curve analysis showed that neither the GLP nor SN had a negative effect on the growth or viability of *V. cholerae* (Fig. 7d). To further test cell viability, AgNPs were also tested against *V. cholerae* at a final concentration of at least twofold and fourfold lower than the respective MICs. SN–AgNPs demonstrated growth and metabolic activity at twofold lower concentrations but with longer duration of lag phase. However, at same dilution GLP–AgNPs demonstrated only metabolic activity but no growth. Growth profiles at fourfold lower concentrations were almost similar to the Luria broth (LB) control which suggested that these NPs concentrations could not inhibit the growth of *V. cholerae.*

The antimicrobial efficacy of AgNPs in the present study was indicated to be due to the combination of their small size (~10 nm) and high surface-to-volume ratio, which allowed nanoparticles to cross through microbial membranes very easily [2]. Our results were in well agreement with the observations reported by Duncan (2011) which suggested that the interaction of AgNPs to the bacterial cell depends on the availability of the surface area, and the size and shape of the silver nanoparticles could play an important role in the enhancement of antimicrobial activity [27]. The antimicrobial activity of AgNPs increases significantly with decreasing size of the nanoparticles, which could be due to the fact that smaller sized particles have larger surface areas for releasing silver ions and can easily pass through the bacterial membrane pores [27]. The exact mechanistic action of AgNPs against microbes is not fully understood. However, several possible mechanisms for the antimicrobial activity of AgNPs have been suggested in literature. It has been proposed that silver nanoparticles can cause cell lysis or inhibit cell transduction. AgNPs could bind to external proteins to create pores, interfere with DNA replication or form reactive oxygen species (ROS) such as hydrogen peroxide, superoxide anions, and hydroxyl radicals.

**Fig. 8:**
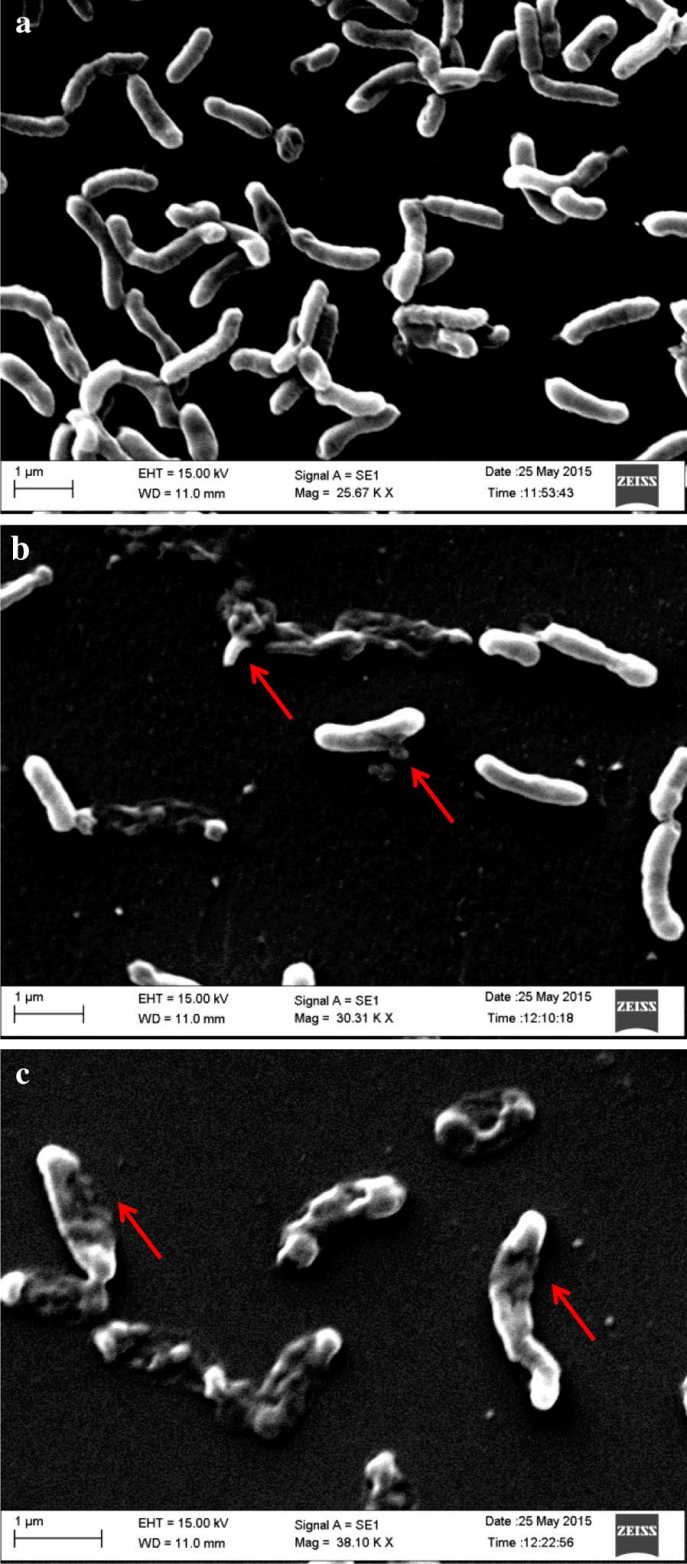
SEM images after 5 h incubation **a** Untreated *V. cholerae* cells **b** GLP–AgNPs treated cells (at two-fold lower concentration than MIC values) **c** GLP–AgNPs treated cells (at MIC value). *Arrows* indicate cell lysis

In the present report, a preliminary study was carried out to understand the mechanism of action of GLP-stabilized AgNPs against *V. cholerae.* For this purpose, the cell morphology of the nanoparticle treated and untreated cells were studied using SEM and results are shown in Fig. 8. The untreated bacterial cells of *V. cholerae* retained their original rod shape and demonstrated very smooth morphology (Fig. 8a). On the other hand, GLP–AgNPs treated cells showed significant changes in morphology with pores and cavities on the cells membranes (Fig. 8b, c). Upon treatment with the nanoparticles, the cells become irregular in shape and size. Moreover, the figure has indicated initiation of cell lysis and disruption of the cell membranes clearly suggesting that the attachment of GLP-stabilized AgNPs on the bacterial membrane might have resulted in cell rupturing. The arrow in Fig. 8 demonstrates the cell lysis and leakage of intracellular contents. It is anticipated that the AgNPs dissipate the membrane electrical potential by disrupting the membrane bilayer, resulting in intracellular ion efflux [1].

These observations were concomitant with the earlier literature reports. Song et al. reported the susceptibility of *V. cholerae* to AgNPs and suggested that antibacterial activity is due to plasmolysis and inhibition of bacterial cell wall synthesis [28]. In another report, the silver nanoparticles are believed to interact with sulfur and phosphorous containing compounds such as proteins on the bacterial cell membrane, change membrane permeability, attack the respiratory chain and cell division machinery, leading to cell death [29].

The antimicrobial properties of AgNPs have been studied in literature, however only very few of those have actually investigated their role in cholera treatment [3, 30]. Jeevan et al. (2011) studied the antibacterial activity against cholera using preliminary agar diffusion assay [30]. An in-depth study of metabolic activity, growth profile and cell morphological changes were not carried out by this group. In a recent report, the effect of silver and zinc nanoparticles against *E. coli* and *V. cholerae* was studied using MIC assay and survival curve [3]. Salem and group reported the synthesis of very large size nanoparticles (90–100 nm) which have small surface areas and cannot easily pass through membrane, and hence are not suitable for biomedical purposes. Moreover efficacy of these nanoparticles was not compared with the antibiotic control. In contrast, the present study demonstrated synthesis of smaller size AgNPs which can easily pass through membrane and cause cell lysis. Furthermore the antibacterial activity of AgNPs was almost comparable to ciprofloxacin and hence AgNPs can be effectively used for the design of novel therapeutic approaches against cholera. Our results were further supported by Morones et al. study which has demonstrated that small size nanoparticles (~1–10 nm) attach to the cell membrane surface and drastically disturb it normal functioning, like permeability and respiration, and they are capable of penetrating inside the bacterial cells and induce further damage by possibly interacting with sulfur- and phosphorus-containing compounds such as DNA [31].

## Conclusions

In the present study, the isolate *O. rhizosphaerae* (ARC-61) was used as a cell factory for the production of silver nanoparticles. Furthermore, the active biomolecule responsible for synthesis and capping of silver nanoparticles was identified and found to be a glycolipoprotein. In fact, the purified exopolymer was successfully used for making suitable smaller size AgNPs and these NPs were stable without agglomeration for more than one month. The prepared GLP–AgNPs have shown remarkable antibacterial activity against *V. cholerae* owing to their smaller size and high surface-volume ratio. The antimicrobial efficacy of AgNPs was more prominent than ciprofloxacin antibiotic. The conjugate was able to rupture the cell walls and cause cell lysis of the pathogen. Thus the present study highlights the possibility of using GLP-caped AgNPs synthesized via *Ochrobactrum* as an inexpensive alternative approach to treat the infection of the *V. cholerae* during in vivo therapy of secretory diarrhea. However, further in vivo biofilm and cytotoxic activity studies against normal cells are required to clearly elucidate its possible application in cholera treatment. Evaluation of AgNPs for in vivo studies shall be the topic of our future research.

## Methods

### Characterization of ARC-61 strain

Genomic DNA extraction, amplification, 16S rRNA gene sequencing and phylogenetic analysis of strain ARC-61 were performed as described previously [32].The identification of phylogenetic neighbours and calculation of pair wise 16S rRNA gene sequence similarities were done using EzTaxon server and aligned using the MEGA software 6.0 [33, 34].

### Green synthesis of silver nanoparticles by culture supernatant

The strain ARC-61 was maintained on agar media consisting of yeast extract (4 gL^−1^), malt extract (10 gL^−1^), dextrose (4 gL^−1^), CaCO_3_ (2 gL^−1^) and agar (20 gL^−1^). A loopful of culture was transferred to the above mentioned media in an Erlenmeyer flask and then incubated in a shaker at 30 °C with a continuous agitation of 200 rpm for 72 h. The fermented broth was made cell free by centrifugation (Sigma^®^4–16 K) at 16,770*g* for 15 min and then filtered through 0.22 µ syringe filter (MILLEX^®^GP). Synthesis of AgNPs was carried out by adding 1 mL cell free SN into freshly prepared aqueous solution of AgNO_3_ (9 mM) in a ratio of 1:9 (v/v). The mixture was incubated at 37 °C in the presence of light.

### Extraction and purification of exopolymer from broth

To gain an insight of mechanism of AgNPs synthesis by ARC-61 strain, EPL was extracted from cell free supernatant by adding chilled absolute ethanol in a ratio of 1:2 (v/v) and kept at 4 °C overnight for complete precipitation. The precipitate obtained was then dried overnight at 80 °C to remove residual ethanol. The crude extracted EPL was purified by overnight dialysis. Dialyzed material was re-precipitated again as described previously [35].

### Characterization of exopolymer

Total sugar content of EPL was assayed by the anthrone method using glucose as the standard [36]. Total protein content in the sample was determined by BCA assay kit using bovine serum albumin as standard [37]. To analyze the total lipid content, EPL (5 mgmL^−1^) was hydrolyzed with 2 % (v/v) acetic acid at 100 °C for 2 h [38]. The lipid precipitated was collected as pellet after centrifugation at 4 °C, 10,000*g* for 20 min, followed by repeated washing with warm water and finally with acetone.

### Antioxidant activity assay

The antioxidant activity of the EPL (or GLP) was evaluated using DPPH radical scavenging assay. The DPPH quenching ability was measured by the method proposed by Zhao et al. with slight modifications [39]. Briefly, 1 mL freshly prepared DPPH solution (0.1 mM in methanol) was mixed with 3 mL of GLP at various concentrations (0.5, 0.75, 1, 2, 3, 4 mgmL^−1^). The mixture was shaken and incubated at 25 °C for 30 min in the dark, and the absorbance of reaction liquid was measured at 517 nm. Butylated hydroxytoluene (BHT) was used as the positive control. The percentage scavenging radical was calculated using the following equation:

$${\text{Scavenging}}\,{\text{rate}}\,\left( \% \right) = {{\left( {{\text{A}}_{ 0} - {\text{A}}_{ 1} } \right)} \mathord{\left/ {\vphantom {{\left( {{\text{A}}_{ 0} - {\text{A}}_{ 1} } \right)} {{\text{A}}_{ 0} }}} \right.} {{\text{A}}_{ 0} }} \times 100$$

where A_0_ is the absorbance of the control (water instead of sample), and A_1_ is the absorbance of the sample.

### Synthesis and characterization of GLP–AgNPs

GLP was subsequently utilized for AgNPs synthesis by adding AgNO_3_ solution to its aqueous solution (4 mgmL^−1^) in 9:1 (v/v) ratio and incubated at 60 °C for overnight. Characteristic surface plasmon resonance of AgNPs was recorded after incubation. Formation of AgNPs was studied by UV–visible spectroscopy by measuring spectra in the range of 300–800 nm using Hitachi double beam spectrophotometer (Hitachi U-2900). Further, the average particle size distribution of nanoparticles was analyzed through DLS technique and the morphology of AgNPs was studied by TEM in JEOL 2100 TEM as described earlier [40]. An elemental analysis of the AgNPs was carried out by a scanning electron microscope equipped with an EDX, which can provide a rapid qualitative and quantitative analysis of the elemental composition of nanoparticles. FT-IR analysis of GLP and GLP–AgNPs was recorded with in wavelength range of 400–4000 cm^−1^ according to the methodology reported by Kanmani and Lim [1].

### Antibacterial activity against *Vibrio cholerae*

The silver nanoparticles were tested for their antibacterial activity against *V. cholerae* EL Tor strain N16961 by the agar diffusion method [1, 41]. The *V. cholerae* was initially propagated at 37 °C in LB at 200 rpm. The overnight grown culture of *V. cholerae* was then again subcultured into LB media for 2 h till 0.01 OD. Subsequently, 100 μL of liquid culture was then spread uniformly onto LB agar plates. Wells of 6 mm diameter were made on agar plates using an agar well borer. 100 μL sample of PBS (pH 7.0), AgNO_3_ (9 mM) solution, both AgNPs (40 μg/mL) were loaded into the wells and the plates were incubated at 37 °C. Zone of inhibition was calculated by measuring the diameter of bacterial clearance after 16 h.

### Determination of MIC by growth and XTT assay

Overnight grown culture of *V. cholerae* was initially subcultured into LB till 0.1 OD. 100 μL samples of this culture were then distributed into 96-well plates followed by the addition of 100 μL of 11 appropriate serial dilutions of AgNPs to each well. The AgNPs were serially diluted with in a concentration range of 40–0.019 μg/mL. GLP and SN alone were also tested, and the ciprofloxacin and LB broth were taken as positive and negative control respectively. The ciprofloxacin was serially diluted with broth at a concentration ranging from 10–0.0048 μgmL^−1^. Plates were incubated for 18 h at 37 °C in orbital shaker. After completion of incubation period, bacterial growth inhibition and absence of metabolic activity were examined by colorimetric XTT reduction assay [25]. 30 μL of XTT (0.5 mgmL^−1^, Sigma-Aldrich) was added to micro-well plate and incubated at 37 °C in dark for 2 h. XTT reduction (reduced formazan-coloured product formation due to metabolic activity of the cells) was measured using microplate reader (Biotek Spectrophotometer, Power wave XS2) at 490 nm [26]. All the experiments were performed in triplicates and average values are reported. Statistically significant differences between the groups were determined using one way ANOVA and multiple pairwise comparison procedures (Holm-Sidak method). Statistical analysis was performed using Sigma Plot (version 13.0) software (Systat corporation, USA). Values of *P* < 0.05 were considered statistically significant.

### Anti-cholera activity of the AgNPs

Growth kinetics studies were performed in 100-well plate using overnight grown culture and diluting it to 1:1000 in LB broth. 100 μL of culture was added with 100 μL of LB broth supplemented with AgNPs or with ciprofloxacin, GLP and SN only. The OD_600_ was continuously monitored after every 30 min in the Bioscreener reader at 37 °C with continuous shaking for 18 h. For growth curve analysis, three independent experiments were performed for each sample. The average values were calculated and reported. Any changes in cell morphology of nanoparticles treated cells were observed using field emission scanning electron microscope (Zeiss Evo 40) as described earlier [42].

## Abbreviations

AgNPs: silver nanoparticles
ANOVA: analysis of variance
BHT: butylatedhydroxytoluene
DLS: dynamic light scattering
DPPH: 2,2-diphenyl-1-picrylhydrazyl
EDX: energy dispersive X-ray analysis
EPL: exopolymer
FTIR: fourier transform infrared spectroscopy
GLP: glycolipoprotein
GLP-AgNPs: glycolipoprotein-silver nanoparticles
LB: luria broth
MIC: minimum inhibitory concentration
PBS: phosphate buffer saline
SEM: scanning electron microscope
SN: supernatant
SN–AgNPs: supernatant-silver nanoparticles
SPR: surface plasmon resonance
TEM: transmission electron microscopy
XTT: 2,3-bis(2-methoxy-4-nitro-5-sulfophenyl)-2H-tetrazolium-5-carboxanilide sodium salt
